# A ‘Control Model’ of Social Media Engagement in Adolescence: A Grounded Theory Analysis

**DOI:** 10.3390/ijerph16234696

**Published:** 2019-11-25

**Authors:** Melina A. Throuvala, Mark D. Griffiths, Mike Rennoldson, Daria J. Kuss

**Affiliations:** 1 International Gaming Research Unit, Psychology Department, Nottingham Trent University, Nottingham NG1 4FQ, UK; mark.griffiths@ntu.ac.uk (M.D.G.); daria.kuss@ntu.ac.uk (D.J.K.); 2 Psychology Department, Nottingham Trent University, Nottingham NG1 4FQ, UK; mike.rennoldson@ntu.ac.uk

**Keywords:** adolescent social media use, adolescent smartphone use, problematic social media use, social media addiction, smartphone addiction

## Abstract

Adolescents actively use social media, which engages them cognitively, emotionally, and behaviorally. However, the underlying psychological mechanisms of engagement have not been adequately addressed. The present study examined adolescents’ psychological processes as these develop in their everyday interactions via social media. The sample comprised six focus groups with 42 adolescents from UK-based schools. Data were analyzed using constructivist grounded theory. The resulting concepts related to individual, social, and structurally related processes, highlighting a synergy between the processes underlying use and a gradual reduction of control as individual, social, and structurally led processes emerge, conceptualized as the ‘*control model*’ of social media engagement. The findings highlight a controlling aspect in engagement and a dynamic interplay between the processes as mutually determining the quality and the intensity of the interaction. Recommendations are provided for examining *control* as a main emotional, cognitive, and behavioral mechanism in problematic and/or addictive social media and smartphone use.

## 1. Introduction

Adolescents benefit from the use of social media applications (‘apps’) and features (i.e., ability to customize content) on major platforms, but empirical evidence suggests a minority also struggle to use them in moderation [1]. The difficulty in controlling frequency of engagement has partially been attributed to the habitual nature of use, and the constant automatic feeding and structural mechanisms of media platforms, which reinforce use [2]. Adolescents experience peer pressure to have a constant online presence to participate in the offline and online environment, to produce content that is likeable and distinctive, and to curate images online in a way that appeals and is acceptable to their peers [3]. Balanced use is inherently difficult to sustain due to individual, social (peer), and environmental factors (i.e., design characteristics), which determine the frequency and severity of the interaction [4]. Growing evidence has acknowledged the impact of persuasive design in initiating and prolonging user engagement [5], similar to the way structural characteristics in games have been associated with a longer duration of play and immersion experience in gamers [6]. In the UK, academic and charity initiatives advocate the development of regulations and a framework to shield young people against the impact of persuasive design strategies (i.e., auto-play, likes, re-tweets), such as the ‘Age appropriate design code’ framework by the 5Rights Foundation [7] and research-driven advocacy projects aiming to promote policies and practices that maximize online benefits while minimizing the harms to children’s wellbeing [8].

Adolescent social media use is associated with major psychological processes underlying use. Psychological processes are operationally defined as cognitive, social, or behavioral mechanisms relating to an individual’s pattern of thoughts, emotions, experiences, and/or behavior, and are critical in increasing the vulnerability for developing a disorder, or are involved in its etiology or maintenance [9], with impacts on perception, learning, language, thought, attention, memory, motivation, and emotion [10]. A process is fluid and transitory, usually running on a continuum, which is modifiable and dependent on context [11].

Main user functions of social media share three crucial processes: Cognition, communication, and cooperation. The sharing of content produces communication and may result in a form of collaborative work within the community of users [12]. These three functions are defined further by social roles and systems (i.e., public and private), which are integrated in personal profiles with different forms of data (i.e., personal or public) and social roles and activities converging, offering self-presentation and surveillance on different facets of life. *Self-presentation, disclosure of personal information*, and *surveillance*—the act of watching and being watched [13]—constitute pronounced processes during adolescence because they facilitate the developmental task of identity formation, which constitute powerful motivational factors [3]. Evidence suggests that apart from intimate, positive, and entertaining updates, self-disclosure fosters more connected relationships [14] and induces more likeness [15], while *supportive interactions* and *social connection* enhance positive affect [16].

Research suggests that user functions may therefore offer positive outcomes. However, usage may also induce processes, which may incur either problematic or pathological engagement for a minority of adolescents [17]. This occurrence could be triggered by habituation, which may reinforce compulsive tendencies and create an environment for the development of problematic conditions or addictions [18]. Other psychosocial factors involved in habitual social media use include *fear of missing out* (FoMO)*, reciprocal liking,* and *social competition* [2]. Studies have found that FoMO mediates the relationship between social media use and online vulnerability [19], and also accounts for a high percentage of variance in social media addiction [20]. Similarly, nomophobia (no mobile phone phobia) has been considered a contributing factor in impulsive smartphone use and the potential for developing social media addiction [21]. Another major process underlying adolescent engagement and the exhibition of *electronic aggression*—defined as aggressive acts enacted in the digital environment, such as mocking, making insulting or threatening comments, spreading rumors [22] —is related to the mechanism of disinhibition and termed the *online disinhibition effect*. This is operationally defined as conscious and subconscious reactions to online experiences fueled by the absence of face-to-face communication and the anonymity or distance afforded by online communication [23]. Mobile communication can facilitate incivility and impoliteness with amplification of content that may elicit hostile communication [24] with little fear of retaliation. This in turn could trigger various levels of overt and covert aggression associated with social media use [25]: From a more nuanced role, such as *trolling* (the act of promoting a pseudo-membership in an online group, with the real intention to disrupt, distract, or trigger tension with the use of inflammatory, irrelevant, or offensive content for amusement purposes [26]) or *banter* (an elusive form of discursive exchanges testing social boundaries, negotiating status, group inclusion, and exclusion [27]) to more severe manifestations of aggression, such as *online harassment* and *cyberbullying*—defined as bullying through digital media “intended to hurt (by the perpetrator) and perceived as hurtful (by the victim); …part of a repetitive pattern of negative offline or online actions; and performed in a relationship characterized by a power imbalance” ([28], p. 499).

Current empirical evidence concerning social media addiction emphasizes uses, antecedents, impacts, and risk factors conducive to social media use (typically via smartphones and often conflated with the concept of ‘smartphone addiction’; [29]). However, there is a scarcity of studies identifying those psychological processes and their interrelationships, which may lead a minority of adolescents from normative engagement to a problematic state of social media/smartphone use. To date, the relationship amongst these processes lacks definition and has received minimal attention in the literature, albeit influencing adolescents’ emotional states. Depending upon their severity and frequency, these processes may act as precursors or as a prodromal state to addictive tendencies. Given: (i) The major neurophysiological and behavioral changes that take place in adolescence [30], (ii) the increase in emotional mental health problems in this age group [31], (iii) the addiction vulnerability [32], and (iv) the technological environment and structural characteristics that are implicated in addiction among vulnerable individuals [2,29], the psychological processes of online communication are crucial in identifying and understanding how a functional and versatile tool like social media may also pose a risk to mental health, and undermine personal wellbeing [33]. In order to identify the mechanisms of how social media use may invoke problematic engagement, it is critical to explore and understand the main psychological processes that are implicated in adolescent online interaction. Consequently, the present study explored these dynamic processes utilizing a qualitative investigation with adolescents.

## 2. Materials and Methods

### 2.1. Design

The present study applied *grounded theory*, a qualitative methodology most appropriate for research concerned with understanding phenomena [34] and producing a conceptual framework of interactions and processes [35]. More specifically, the study employed *constructivist grounded theory* [36], which is particularly appropriate for the present study because its relativist epistemology allows for co-construction of theory and meaning by researcher and participants [37], while studying processes and linking the individual with the social context and encouraging a deep analysis of the phenomenon [38]. This epistemological approach was particularly relevant in the present study because it attempted to co-construct the underlying psychological processes of social media use and the phenomena online. Problems and concerns with social media interaction were explored to: (i) Decipher the psychological processes underpinning use and, (ii) generate novel theory regarding the interrelationships between the processes and their association to problematic and/or compulsive use. At the commencement of the research, there was no substantive theory to explain the nature of processes in social media use, and theory generation was therefore a highly appropriate outcome for the proposed study. The present study examines psychological processes (cognitive and emotive) associated, influenced, and/or facilitated by social media use. Additionally, it highlights the ways these processes are interrelated and influenced by group dynamics and the media environment itself and how these may be implicated in potential negative impacts of social media use during adolescence.

### 2.2. Participants

Participants (*N* = 42) aged 12 to 16 years (*M* = 13.5 years, *SD* = 2.3) were selected in collaboration with three local secondary schools in the East Midlands area of the UK, including a mix of an all-female school and two co-educational schools. Students were primarily white (63%), black (22%), and Asian (15%), with an almost even gender split (48%/52% female/male), and from diverse socio-economic communities: Upper socio-economic class (20%), middle class (54%), and lower class (26%). The study targeted adolescents due to the: (i) high online usage this age group exhibits, and the vulnerability to peer evaluations and risk behaviors [39], (ii) heightened vulnerability to excessive online use, leading potentially to addictive symptoms [40], and (iii) development of body-image concerns and an overemphasis on peer comparisons that may be associated with the development of eating disorders and obesity [41,42,43].

### 2.3. Procedure

Ethical approval for the study was granted by the research team’s university Ethics Committee (No. 2017/109). A semi-structured focus group guide was developed based on key perceptions of concerns and psycho-emotional problems arising from the various uses of social media. Given that the sample was adolescents, it attempted to elicit processes involved through the main social media uses, concerns, incidents, and experiences arising from use followed by perceived impacts. Data were collected from six adolescent focus groups from three schools in the East Midlands area over a period of two months. Upon agreement for participation, information sheets about the nature of the study were distributed electronically from the school administration to the parental community, along with parental opt-out forms. The sign-up for participation was conducted by the schools’ administrators and there was no compensation or reward provided for participation. Each focus group lasted 60 to 100 min, and was audio-recorded and transcribed verbatim with the use of NVivo 12.00 software. Adolescents were asked to discuss specific uses and practices, motivations for use, and any problems, concerns, and intervention needs.

### 2.4. Data Analysis

Coding took place initially using open coding, followed by axial and selective coding. The analysis was conducted simultaneously to coding the data, starting off with concepts, which were the basic units of analysis and gradually built up as new data emerged with repeated concepts grounded in the data. For example, one participant mentioned: “*Some people post anything, and they don’t care, to be fair. I wish I could be more like that…and I don’t judge them for that, I am just overthinking…*” (FG2F4). This was labelled “*preoccupation*”. Subsequent similar iterations were compared to the initial incidents and through the process of constant comparisons formed categories, such as ‘*cognitive salience*’. These categories were higher in level and more abstract and formed the building blocks for the theory development and integration. These categories were then formed into higher order categories with more abstract headings based on their functions and interactivity with the other processes. Memo writing was constantly updated as the analytical process evolved. Theoretical sampling was met through the meaning the adolescents attached to those interactions and examining representativeness and regularity of the categories in the subsequent focus groups. Hypotheses about relationships amongst categories started to be formed and checked against new data, and broader structural conditions emerged and were integrated in the analysis (structurally-led processes) until reaching saturation [36]. This continuous and systematic process of data collection and analysis allowed a comprehensive construction of a theoretical formulation of inter-related processes taking place when adolescents engage in communication on social media. Participant identifiers were constructed by the number of the focus group they participated in, their gender, and their participant number.

## 3. Results and Preliminary Discussion

The study’s findings highlighted several complex and inter-related processes underlining adolescent social media engagement and embedded in adolescent social media user functions, comprising three types of processes (Table 1): (i) Individual, conceptually divided into *cognitive and emotional* processes and termed as ‘*engagement to control*’ content, relationships, and self-presentation; (ii) socially-constructed, termed as ‘*controlling the relational self*’; and (iii) structurally led processes (driven by the platforms’ designs encouraging a specific repertoire of behaviors), termed as ‘*hooking and hunting*’. Social processes were based on group responses and were empowered by group dynamics, whereas structurally led processes were platform-related mechanisms aiming to reinforce engagement.

### 3.1. Individual Processes

#### 3.1.1. Cognitive Individual Processes: ‘Engagement to Control’

*Heightened self-consciousness, cognitive salience, and vigilance*. Students reported experiencing a state of higher self-consciousness (awareness of oneself and actions) and alertness (“*Makes everybody a lot more self-conscious*” (FG2F6)), instilling arousal as a result of their presence and engagement in social media. Participants attributed this to the photographs and content that adolescents posted and to expectations for feedback to their posts, either in the form of ‘likes’ and/or comments. This elevated state of preoccupation with personal appearance was viewed as magnified via the photo-sharing culture of platforms, and judgmental peer attitudes on social media leading to a constant need for enhancement of photos and content. Additionally, peer expectations for instant availability created pressure for checking. Leaving notifications or messages unattended was perceived as a sign of being ignored, which created communication complications. Adolescents therefore reported experiencing a state of constant salience and vigilance for new content: “Users’ permanent cognitive orientation towards online content and communication, as well as their disposition to exploit these options constantly” [44], p. 1.

*Exhibitionism, social comparison, and appraisal*. Adolescents exhibited specific friendships on social media as evidence of time spent together or as a way to garner support and approval from peers: “*They do it to show how close you are and that you have a funny relationship but at the same time it might be embarrassing for the person*” (FG4F3). This was often conducted by contemplating peers’ considerations regarding a physical feature, while others chose to disregard others’ perceived insecurities for appearance when posting a group photograph, which was a common cause of concern and misinterpretation amongst peers. Therefore, a state of arousal and overthinking was expressed also about the level of self-disclosure. Similarly, the practice of sexting—the act of sending and/or receiving sexually explicit texts and images [45]—was often shared without the knowledge or agreement of the individual depicted, which was referred to as another common practice amongst male adolescents particularly. Social comparison is a process of comparing oneself with others in order to evaluate and to self-enhance [46]. Adolescents engaged in constant self-comparison and critical evaluation of others’ social media practices, and was viewed as a harsh practice that all adolescents were involved in: “*It’s like you scroll through Instagram and automatically I don’t mean to be but I’m like the most judgmental person*” (FG2F1). To balance out the highly critical environment of the more public social media platforms (i.e., Instagram), relational closeness was sought through exchanges of intimate day-to-day experiences that were free from social comparison and scrutiny.

*Distractibility and procrastination*. Distraction refers to the removal of attention away from a negative situation to a neutral or positive one and is considered a cognitive strategy to regulate emotions [47], which may be adaptive or maladaptive, depending on whether it is combined with acceptance or avoidant strategies [48]. A majority of adolescents referred to the constant distraction experienced from major tasks (i.e., homework) attributed to: (i) External interruptions due to notifications or direct messages: “*When I’m doing homework, it is very distracting, one question might take me like an hour, because I chat on my phone*” (FG1F1); or (ii) internal interruptions, in the form of preoccupation with (and expectations to) receive comments and messages: “*So even if I put it in my bag say if I have Maths, then I am like: Oh, maybe that person might be texting me, I am going out to check my phone*” (FG6F3). This mental shift was viewed as impacting focused engagement in schoolwork and as a detrimental process in social media engagement. Additionally, adolescents experienced feelings of procrastination, using social media to delay tasks, “*Snapchat it is quite an easy way out, it’s accessible for people who want to procrastinate a bit*” (FG4M2) and viewed as influenced (at least partially) by their ability to control their distractibility.

#### 3.1.2. Emotional Individual Processes: ‘Engagement to Control’

*Relational closeness for social facilitation*. Students reported a need to have both a public and a private discourse online with Instagram as the main public platform and ‘*Finstagram*’—a second more private Instagram account and Snapchat to serve as platforms for the exchange of intimate, informal moments and experiences amongst an inner circle of friends: “*…people now have created second accounts, because they are so self-conscious*” (FG2F3). In social situations, adolescents experienced social facilitation in two ways: (i) Using social media as a means to overcome social discomfort or social anxiety by replacing ordinary moments of waiting (e.g., on the bus), and (ii) as offering a boredom-reducing solution when in the presence of ‘unwanted’ others.

*Separation anxiety to fear of loss of self-control*. Adolescents experienced anxiety and negative mood states when not having their mobile devices, when unable to contact or access content (nomophobia). Self-control was viewed as compromised due to FoMO and the constant pressure for availability. Adolescents preferred to keep devices physically away during homework (“*I made my brother hide it while I was revising. It was so like addicts*” (FG2F4)), yet they experienced a difficulty resisting attending to notifications or direct messages. A difficulty was also expressed where sleep time routines were violated, and sleep compromised either due to reading notifications or purposeless scrolling through feeds.

*Mood modification*. Adolescents experienced volatile emotions as a result of online interaction. Mood was often dictated and altered by (i) negative peer responses, (ii) inability to access devices, and/or (iii) feeling unable to communicate with friends (i.e., “*What I do is when someone says something and uses sarcasm, I just feel okay but if they keep on saying it, especially, if it’s my friends, I just take it hard and then I need to calm down, it does have an emotional impact*” (FG5F5)). However, participants also reported an inflated sense of self and entitlement for liking and approval in relation to self-presentation. The curation of personal identity online was raised as a critical daily task, fulfilling users’ needs to gain acceptance and enhance one’s social status within the online community. Therefore, in response to the increased effort to identify, upgrade, and post the preferred image were reciprocal expectations for liking and approval. However, the practice of enhancement often took extreme forms beyond identification in real life, viewed as causing disillusionment, distrust, and higher aggression as a result.

### 3.2. Socially Induced Processes Online: ‘Controlling the Relational Self’

Adolescents also discussed processes they experienced as a result of belonging to various online groups. These processes were identified as: (i) *Deindividuation of self to conform to group norms*, (ii) *diffusion of responsibility and ensuing social disinhibition*, (iii) *relational aggression on the continuum*, (iv) *interpersonal surveillance and mirroring*, and (v) *social disruption*.

*Deindividuation of self to conform to group norms*. Loss of personal will and the tendency to follow and agree with other in-group opinions or actions was experienced by adolescents as either passive tolerance or indifference. Peers often expressed succumbing to the common spirit of the moment despite personal disagreement (i.e., “*It’s like a trend other people are doing it so if they want to be with those people they’re trying to do it themselves; they might not necessarily think it’s a pretty good thing to do, but since other people do it they do it themselves*” (FG3F5)). To an extent, this process was facilitated by conforming to group norms and by passively tolerating bad behavior through inaction, indifference, and/or aggressive acts. Adolescents experienced a high degree of group influence in group chat situations, where peers tended to exhibit social desirability rather than supporting a peer who was in distress: “*In group chat, if someone says something, it’s a lot easier for people to agree with them rather than back the other person up: If they tell you,* ‘*you’re fat*’ *or whatever, then it’s a lot easier to agree, rather than saying—that’s not right!*” (FG4M2).

*Diffusion of responsibility and ensuing social disinhibition*. Adolescents experienced a loss of responsibility “*I think on social media it is a lot easier to physically move your fingers and type in words and actually say something to someone because you can write anything but would you say that on their face?*” (FG4M1) and conforming to the group dynamic expressed as inactivity or tolerance of bad behavior towards other peers, “*It’s easier to say anything, you’re more brave online, not scared*” (FG5F1).

Diffused responsibility stemmed from group dynamics and perceived peer pressure (fueled by a degree of cyberstalking [49]) and a lack of awareness of the impact of consequences of online actions on others: “*I wanna look at what other people are doing…When you don’t want to talk in person, people would say things they would not dare say to their face, and they do it online*” (FG2F2). Adolescents viewed the absence of face-to-face communication and facial expression as facilitating uncivil ways, causing considerable distress to the recipients of the messages. Social disinhibition was experienced as a great degree of freedom online and a greater sense of confidence and feeling less fearful online, without consideration for the consequences: “*They can’t see you, can do whatever you want to, it feels like there is less consequences, whatever you’ve typed…because they’re behind a screen. You’re shielded*” (FG3M4). Social disinhibition was viewed as taking different forms and was expressed in various degrees of severity with behaviors online perceived at times as evolving into some form of cyberbullying “*And even if you have an argument with one friend, you can just screenshot it and then there is so much going on:* ‘*why did you screenshot it?*’*—they send you the argument, the text messages, people are more touchy finding out things on social media*” (FG6M2). “*Some people text you, who you don’t even know and judge the pictures that you have posted. But you just say something back and block them, just like,* ‘*Bye you are blocked*’ *but sometimes it can get quite nasty, like bullying*” (FG5F5). Lack of physical cues or facial expressions led to an almost automated mindless empowerment and taking the step to articulate a negative comment, not shared otherwise in a face-to-face conversation: “*The fact that you don’t see them if you are talking about a problem, you can’t see them and is easier and you can say something mean that you would not say to someone’s face*” (FG1F1). Misinterpretation of intention escalated to larger issues and a spiraling of events magnified out of proportion, spilling over to offline relations and vice-versa. Additionally, feeling forced to participate socially was viewed as another source of frustration, which activated social disinhibition. *Relational aggression on the continuum*. The expression of hateful comments was a common adolescent experience, “*With social media people don’t quite know if you are saying a joke because they don’t see the sarcasm, something might start up as a joke it might spiral into other things and people might end up hating each other with just a few words on Instagram. It happens quite often*” (FG6F6). The online environment was viewed as facilitating the exhibition and attraction of relational aggression. Being able to freely express themselves without face-to-face contact facilitated harsh attitudes towards others. Such behavior appeared to be observed more on specific content transient platforms (i.e., Snapchat), which reinforced the degree of indifference or aggression: “*Especially with Instagram, there have been certain circumstances where people can be very manipulative or bullying…people would say things they would not dare say to their face*” (FG5M7). In the case of fallouts, adolescents criticized the practice of screen-shooting the arguments and sharing the screenshots amongst friends. Online sext-shaming was another expression of relational aggression. Sexting images with inappropriate content was viewed as a frequent practice for adolescents with an emotional impact: “*People do things that shouldn’t do. People texting other people and screenshots and sharing again, it happens quite often*” (FG2F6); “*It feels horrible, when you give someone trust and then they back down on that trust –it really upsets you*” (FG2F3). Another form of social disinhibition was ‘phubbing’, which has been operationally defined as a persistent engagement in smartphones by checking emails and social networks, playing games, listening to music, or other activities as a way of avoiding face-to-face communication [50]. Adolescents employed smartphone use to ‘phub’ unwanted or less accepted peers by persistent engagement with their smartphone.

*Interpersonal surveillance and mirroring*. Surveillance of peers’ activities online was acknowledged as a common practice related to FoMO: “*I’m very nosy, it’s a way to see what others are doing and get some feedback on what is going on*” (FG6M2). Social media celebrities and other famous individuals were viewed as influential amongst the teenage population: “*Those people you follow, you see their hobbies, interests, fun videos and you end up spending loads of time on it*” (FG3M5).

Mirroring influencers’ body images and setting goals based on these standards was common yet was contradicted by perceptions of unrealistic and unhealthy standards of beauty, which were being promoted online. Despite the initial enthusiasm expressed about the ideal standards, adolescent participants advocated against the promotion of unhealthy practices of individuals promoting negative mental health and peers who mirrored unhealthy types of views, behaviors, and appearances, “*I think with celebrities and models, when posting a selfie and they put their body and we think it looks amazing! so we are like OMG, I need to get my body like that, but this is wrong*” (FG3F2). Additionally, adolescents expressed concerns that private actions enacted online may arrive in the public domain, with ensuing feelings of embarrassment and shame.

*Social disruption*. Adolescents experienced peers as being antisocial in offline social situations (i.e., school breaks, parties) as initial peer gatherings were eventually turned into online interaction in the presence of others, “*These people are all on their phones, they are unsocial, but that happens a lot …it is cutting up the social aspect of life*” (FG1F6). This differed from phubbing because it was not an intentional act to avoid an unwanted peer. Social disruption incurred due to the common practice amongst adolescents in many offline social situations to drift off and eventually withdraw to their smartphones, exhibiting a preference for online interaction, despite the initial interest to interact. This practice was extended to school break-time or other social instances, with peers exclusively engaged with their smartphones, disregarding the presence of schoolmates or friends. Disruption was also being experienced by distraction and the inability to impose self-control by ignoring smartphone notifications. Overinvolvement with smartphones was considered a disruption of naturally occurring social interaction and lack of balance: “*I think people are trying to have a balance between social life and their social media life*” (FG4F3).

### 3.3. Structurally Induced Processes: ‘Hunting and Hooking’

‘*Hunting*’ was experienced during the acquisition of strategies to achieve a higher reward potential, such as actively seeking to maximize opportunities for reward and reinforcement, while ‘*hooking*’ entailed strategies which endorsed habit-forming behaviors and processes among adolescents.

*Habituation, automaticity, limited time content, and psychological investment*. Adolescents experienced social media use on their smartphone as an automatic process with constant smartphone checking. Use was not ceased even during school lesson time, homework, or sleep routines. Adolescents reported always carrying their devices with them, except for mealtimes due to family or school rules out of fear that the device might be taken away. This habitual use was associated with a state of high self-consciousness and preoccupation regarding new interactions and/or new content. Additionally, if unable to check or perform specific routines (i.e., Snapchat streaks—number of consecutive days of active interchange of photos amongst two friends on the platform [3]), adolescents experienced negative emotionality. Content available for a limited time only as in Snapchat encouraged further online disinhibition: “*Also, especially on snapchat, people can say stuff and think that it’s okay because it doesn’t save and even if you have an argument with one friend you can just delete it*” (FG5F8). Adolescents experienced psychological investment and a bond with their peers on social media, and no adverse effects would motivate them to give this up: “*Go to check my streaks first thing in the morning so I don’t lose them. Nothing comes in the way of having your streaks, your life depends on them, maintaining those streaks*” (FG5M2).

*Triggering activation and anticipation of reciprocation*. Adolescents often expressed their social media use having been triggered by environmental cues (i.e., social media notifications): “*I find it quite hard when somebody messages you and it is personally directed to you, it leads you to open it and then get distracted. It is a temptation*” (FG3F1). Expectations of instant attendance to notifications was expressed as a key behavior, which led to a constant state of alertness. Not answering back and delaying reciprocation was experienced as a sign of ignoring and neglect by the other individuals and viewed as requiring an apology. Cue activation was also internal and hypothetical, denoting preoccupation about inability to instantly interact, “*So even if I put it in my bag say if I have Maths, then I am like: Oh, maybe that person might be texting me, I am going out to check my phone*” (FG6F3). Similarly, an inability to reply instantly was associated with feelings of anger, agitation, and/or compulsive tendencies.

*Reward and reinforcement seeking*. A key process for adolescents was reward-seeking in the form of followers, ‘likes’, and/or ‘streaks’. Adolescents devised strategies to increase their popularity levels (“*If I’m with a friend that doesn’t have many friends, I don’t get many likes, but if I am with a friend that’s got loads of friends, then I get loads of likes as well*” [FG6F5]), and photos judged as not good enough determined adolescents’ emotional state and state of self-confidence. Enhancement and enrichment were experienced by adolescents due to novelty, innovation, and variability in social media, which further reinforced engagement. Novelty in platform content was considered a positive feature benefiting and providing adolescents with increased opportunities for exposure (i.e., daily news feeds). This practice differed from any other traditional media but was viewed also with some skepticism due to its questionable quality. Fake news or poor quality of news was normalized as part of the reality of exposure to social media.

*Wanting more and tolerance*. Higher reward-seeking led adolescents to constant strategies to correct and upload new photos with higher ‘approval’ potential. The number of friends was a direct measure of popularity “*People think like if you’ve got more followers then you get more likes because more people will follow you*” (FG2F2) and being associated with peers with a higher number of friends was considered a successful strategy to reach a higher number of likes in their uploaded photos. Another strategy used was the application of enhancement techniques (i.e., make-up and filters). However, use of filters was perceived as reaching hyperbolic levels, altering appearance to the extent of deception. Additionally, adolescents perceived a discrepancy between what companies defined as the norms of beauty and what adolescents decided to promote. Social media were perceived as an arena where although various possibilities of norms can co-exist (i.e., thinness vs. plus size representation), ideal image standards of beauty dictated adolescents’ choices. This led to a constant negotiation of reaching the ideal versus the normal, and reinforced interaction: “*And it makes you want to change yourself. If for example, I shade it differently, and I put something different from what I last put on, I don’t get as many likes, so clearly, I’m not good enough. Makes your self-confidence go down. You wanna take another one and try to make up for it*” (FG4Μ5).

## 4. Discussion of the Emergent Model

**Figure 1:**
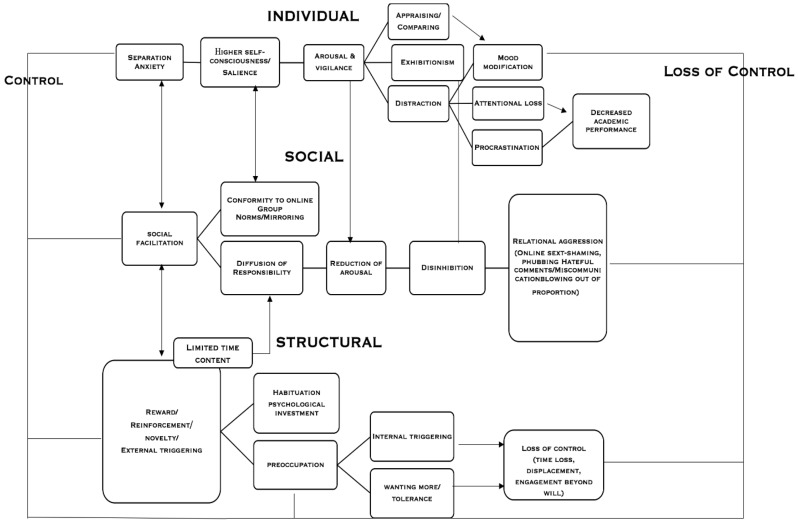
The ‘control model’ of engagement.

The psychological processes evidenced comprised three core categories and formulated a theoretical model termed ‘the control model’ of engagement, highlighting control mechanisms and processes on three levels involved in social media engagement (Figure 1): Individual processes occurring at an intrapersonal level, socially-induced processes forming via social interaction, and structural-level processes evoked from platform design deliberations, which influenced adolescent engagement levels. Findings corroborated a dynamic transition from a state of initial controlled engagement to define content, relationship formation and self-presentation, driven by anxiety mechanisms (anxious preoccupation, salience and vigilance, fear of peer evaluations, exhibitionism) to a reduction of control initially with a loss of attentional focus and of time spent online with implications for academic achievement. Loss was further experienced through group-led dynamics of deindividuation and/or conformity: Group processes (i.e., disinhibition and mirroring) led to either submission to group norms or deindividuation and diffusion of responsibility. This resulted in lessening the degree of personal responsibility and allowing for more aggressive relational phenomena to take place. Aggressive responses may be further intensified by separation anxiety (FoMO and nomophobia) and fear of social exclusion, which reduces sense of control and ability to respond [51].

Structurally induced processes (i.e., *reward* and *triggers*) further reinforce individual processes (i.e., *cognitive salience* and *vigilance, distractibility*), and increase opportunities for social processes to take place (i.e., *interpersonal surveillance, mirroring*, and *social disruption*). Continued use was encouraged and facilitated through habituation, reinforcement, and further investment in the medium, content, and relationships. Therefore, loss of control was experienced at an individual level with a gradual change from a self-referential state and preoccupation with controlling perceptions of personal identity and representation online, to control of group interactivity with structural processes partially determining, facilitating, or reinforcing psychological and emotional outcomes. For example, the process of preoccupation appeared to lead to higher online vigilance, which could be related to checking and habitual enactment, associated in the literature not only with time investment but with psychosocial problems (i.e., anxiety, depression, and loneliness) [52]. Depending on how salient and intensive these processes are within the individual, they may exacerbate potential negative impacts of use (i.e., escalation to higher relational aggression) and may dictate the interpersonal (i.e., relationships with peers) and the intrapersonal (i.e., self-concept) context of the adolescent and lead to potential psychopathological symptoms (i.e., anxiety and/or compulsive use).

Therefore, the emergent ‘*control model*’ of engagement identified: (i) The inter-relationships between individual, social, and structurally induced processes in defining social media engagement; (ii) the gradual transition from a state of individual controlled use to a state of reduced control through the interaction of processes, determining attitudinal and behavioral outcomes; and (iii) control of self-concept, content, and relationships as a principal agent of online engagement—which goes beyond uses and gratifications in social media use.

On an individual level, the findings highlighted separation anxiety as a psychological process underlying adolescent user engagement associated with (i) an increased state of *self-preoccupation* with adolescents’ online identity, (ii) *cognitive salience* of the online environment, and (iii) *vigilance* in adolescents [44]. The extant literature suggests that negative psychological and physiological outcomes are associated with smartphone separation and the inability to answer calls during cognitive tasks [53,54]. *Social comparisons* and *fear of critical appraisal* and evaluation by peers also led to *heightened self-consciousness*. Preoccupation with online identity signaled a state of constant alertness and arousal manifested in more frequent and intense engagement, which appeared to interact with *cognitive salience* and *vigilance* of the online content [55]. It is plausible that both processes are associated with constant checking [44], decreased wellbeing, and reduced mindfulness [56], and with parental reports of hyperactivity/impulsivity, anxiety, depression, loneliness, and FoMO in children [57].

Additionally, checking behaviors appear to have a cognitive impact due to the repeated external interruptions and attentional micro-disengagements or internal interruptions due to vigilance, leading to *distractibility* [58]. *Distraction* is an emotion regulation mechanism of moving attention from negative emotions to non-negative issues [59], and a more adaptive coping strategy in reducing depressive mood relative to rumination [60]. Despite its regulating function, distraction produces an attentional conflict (offline vs. online, platform switch), causing arousal and effort for attentional focusing leading either to *facilitation* [61,62] or shallow processing with impacts on productivity and academic achievement [63].

The present study’s findings highlight the existence of social processes relating to a negotiation of control due to group dynamics. This involved the transitioning from a state of controlled engagement to a gradual loss of control within online group membership. This model of engagement draws from two psychological models with their key processes of *deindividuation*, *social facilitation*, and *diffusion of responsibility*. First, *social facilitation theory* [64] explains how (in the present study) the online presence of others may energize online performance and encourage diversion from offline tasks. Second, *deindividuation theory of aggression* [65] denotes how being part of a group facilitates the release of inhibition with ensuing diffusion of responsibility. Overall, the presence of others induces social facilitation and increases arousal, while reducing responsibility, self-awareness, and accountability [64,65]. External attentional cues and external stimulation from peers or social media feeds gradually induce deindividuation because they distract from internal thoughts and rumination [60], while reduced self-awareness or preoccupation with self-concept or impression management [66] reduces control and may disinhibit aggression [49,67].

Alternatively, adolescents perceived users exhibiting greater conformity to social norms, despite personal disapproval, which may act congruently or antagonistically to deindividuation, and possibly reduce antisocial tendencies [68]. Additional group processes, socially facilitated and amplified by diffusion of responsibility, were related to *aggression* (i.e., *online sext-shaming*) and *social disruption*. This confirmed evidence highlighting increase in adolescent loneliness [69]. An *inflated sense of self*, which emerged as a process afforded by social media, might contribute to impulsive behaviors, potentially in the form of more aggressive interactions. The present findings highlight that *relational aggression* online ranged on a continuum from online disinhibition to various forms of cyberbullying, potentially implicated in psychopathology (e.g., [70]). As an example, *phubbing* has been found to reflect a dependency on smartphones and to be associated with smartphone addiction [71] and problematic social media use [72,73].

Social comparisons and appraisals were other social processes, which appeared to partially underlie cognitive preoccupation and vigilance with potential mental health impact [74]. Negative *social comparisons* on social media negatively affect mood, emotions, appearance, and physical health perception [75,76], disordered eating [77], and mediate wellbeing [78]. The use of ‘Finstas’ was reportedly an alternative way to mitigate peer pressure for ideal online presentation within the platform, which is prevalent in the main Instagram account [3]. Related to self-presentation was the process of *exhibitionism*, manifested through (i) showcasing high-profile friendships—boosting the reputation of the adolescent amongst the in-group—(ii) sexting, and (iii) sext-image sharing. The photographic image was evidence of time spent together with socially influential peers and uploading became a continuous act. This process may be underlying a behavior that has been termed ‘*selfitis*’—the constant act of selfie-taking with its compulsive nature [79,80]. Sexting appears to be an increasingly common practice amongst adolescents, with a mean prevalence for sending and receiving sexts in one study of 14.8% and 27.4%, respectively [81]. Additional processes included *mirroring of behaviors of influential others* and *interpersonal surveillance* confirming increased engagement [82] and a contribution to body dissatisfaction issues [83].

The present study’s findings also highlight the emergence of platform-related, structurally induced processes, and identified two types of mechanisms depending on their function. Social media activities per se have been suggested to be potentially addictive [84], with embedded structural mechanisms that reinforce prolonged engagement and addiction vulnerability [2]. ‘*Hooking*’ processes (i.e., cue-activation, anticipation of reciprocation, limited time content, and psychological investment) aim to attract and retain adolescent attention and engagement through mechanisms encouraging habituation and constant checking [2]. For example, ‘reciprocal transparency’ (awareness if a notification has been viewed or screenshotted, etc.) is a structural characteristic that adds transparency to the online communication, yet if unattended or ignored may cause ‘response latency’ [85], potentially reinforcing preoccupation and checking, thus endorsing a more habitual engagement with continued usage becoming less goal-oriented and performed without a purposeful cognition [86].

Additionally, adolescents experienced a state of *psychological investment* in social media activity and devices, which provide access to content and are related to separation anxiety [53]. Psychological investment appeared as a related process in studies reporting body and facial dissatisfaction [87], and increased browsing and depressive mood [88]. Excessive investment may reflect compulsive tendencies in managing the relational self. ‘*Hunting*’ processes (i.e., reward-seeking) comprised active manipulation for higher reward acquisition, triggered primarily by the variable reward reinforcement similar to gaming, acting as potential antecedent to problematic use due to reinforcement sensitivity [6,89] and neurobiological activation in neural regions implicated in reward processing, social cognition, imitation, and attention [90]. However, behavioral reinforcement factors have not been sufficiently studied to date [91], particularly in relation to their interaction with individual and other situational factors.

The associations proposed were grounded within the particular set of data, which highlighted an indicative pathway. However, given the heterogeneous nature of use, these could not be considered exhaustive or comprehensive as they depend on a host of factors, which could influence outcomes differentially and may not be addressed within the framework of this research study. Additionally, the model can neither prescribe how transient or longer lasting these processes are nor their specific impact on adolescent mental health. Limitations of the present study also pertain to issues of generalizability due to the study’s exploratory nature using small homogeneous focus group samples. Additionally, self-reporting concerns and problems may be inaccurate due to social desirability and selective memory.

The present study’s theoretical and prevention implications are important in terms of understanding problematic processes and embedding mechanisms to control them either by substituting them with positive ones or stopping their escalation. The theoretical implication refers to mapping inter-related processes, which may lead to problematic or addictive social media use and adds to understanding a process-oriented model of problematic social media use, which accounts for a more systemic view of processes. This highlights the interplay between the individual and the situational environment, which has been overlooked in the cyberpsychology literature. In terms of practical implications, one key research area recommended to further investigate is relational aggression, which may be addressed by interrupting the process of deindividuation and promoting empathy and personal responsibility online or by providing information on how to respond to harmful social media content effectively and foster reinforcement for prosocial behaviors and support provision to others. Additionally, the study provides insights that may be embedded in media education, such as conflict-resolution skills and perspective taking [92]. Further studies could examine the current model and delineate the relationships between these processes in a quantitative manner and establish associations with user experiences, dispositional traits, and situational characteristics within the social media environment, and provide clarity in terms of mental health impacts and long-term effects.

## 5. Conclusions

The findings of the present study highlight the existence of control mechanisms and processes involved in social media engagement, which led to a theoretical model defined as the ‘*control model*’ of social media engagement. The model emphasizes the psychological process of control as a key mechanism, moving beyond uses and gratifications, and designates a gradual transition from a state of controlled engagement at an individual level, into a potential gradual loss of control when the social and structural processes come into interplay. Additionally, it describes the interrelationships between the three levels of processes, which may take on different power positions depending on the relation between the parameters and the level of engagement. Based on the current literature, these major psychological functions merit further investigation, as given a conducive context (i.e., a trauma or a hostile family or peer environment) may act as antecedents of or risk factors for problematic smartphone use for a minority of individuals. Depending on the quality of engagement, the meaning attached to the interaction, and the frequency of use, a minority of adolescents may be predisposed to engage in social media use in a problematic or addictive way.

Equally, the above study highlighted an emerging area of research—the importance of design mechanisms within the social media environment, which play a significant role in the initiation and maintenance of online engagement. More research should be encouraged to understand how persuasive design elements capture young peoples’ attention and reinforce both active and passive social media use. Critical to this endeavor is the engagement of young people in the research process and in providing them with a voice that raises their concerns and promotes their recommendations for improving their online experiences [5,93]. This could in turn be translated into effective policy making and intervention by social media operators. More specifically, the identification of these design elements can help in (i) prevention by educating young people on how platforms and tech designers endorse habitual and problematic use, policymaking (by prohibiting potentially exploitative design characteristics), and theory-building (by demonstrating ways that specific persuasive design elements are implicated in problematic use). The recent announcement by Instagram to test globally the banning of likes on its platform, by temporarily disguising them from its users, is a long-awaited corporate response to the growing concerns regarding the impact of social media use on young people’s mental health [94]. However, unless access to user data for research purposes is granted by social media platforms, providing researchers with behavioral tracking data following the gambling industry’s example [95], research findings will still be presented with methodological limitations and arguable associations between user behavior and potential harms. Therefore, public policy, educators, and parents should advocate for a more transparent and socially responsible industry approach, which would reflect genuine interest in the protection of young people’s rights in a rapidly evolving digital environment.

## Figures and Tables

**Table 1:** Individual, social, and structural processes in social media engagement.

**Concepts**
**Individual—Cognitive Processes: ‘Engagement to Control’**
▪ *Higher self-consciousness, cognitive salience, online vigilance and arousal* ▪ *Distractibility/procrastination* ▪ *Social comparison and critical appraisal* ▪ *Exhibitionism*
**Individual—Emotional Processes ‘Engagement to Control’**
▪ *Relational closeness* ▪ *Separation anxiety to fear of loss of self-control* ▪ *Mood modification*
**Social Processes: ‘Controlling the Relational Self’**
▪ *Deindividuation of self and conformity to group norms* ▪ *Diffusion of responsibility and ensuing social disinhibition* ▪ *Relational aggression on the continuum* ▪ *Interpersonal surveillance and mirroring* ▪ *Social disruption*
**Structurally Induced Processes: ‘Hooking and Hunting’**
▪ *Habituation, automaticity, novelty, limited time content* ▪ *Triggering activation* ▪ *Preoccupation with constant checking and anticipation of reciprocation* ▪ *Psychological investment* ▪ *Reward and reinforcement seeking/Wanting more and tolerance*

## References

[1] Kuss D.J., Griffiths M.D. (2011). Excessive online social networking: Can adolescents become addicted to Facebook?. Educ. Health.

[2] Griffiths M.D. (2018). Adolescent social networking: How do social media operators facilitate habitual use?. Educ. Health.

[3] Throuvala M.A., Griffiths M.D., Rennoldson M., Kuss D.J. (2019). Motivational processes and dysfunctional mechanisms of social media use among adolescents: A qualitative focus group study. Comput. Hum. Behav..

[4] Griffiths M.D., Lopez-Fernandez O., Throuvala M.A., Pontes H., Kuss D.J. (2018). Excessive and Problematic Use of Social Media in Adolescence: A Brief Overview.

[5] Creswick H., Dowthwaite L., Koene A., Perez Vallejos E., Portillo V., Cano M., Koene A., Vallejos E.P. (2019). “… They don’t really listen to people”: Young people’s concerns and recommendations for improving online experiences. JICES.

[6] Griffiths M.D., Nuyens F. (2017). An overview of structural characteristics in problematic videogame playing. Curr. Addict. Rep..

[7] 5Rights Foundation What We Do. https://5rightsfoundation.com/in-action/.

[8] Livingstone S., Third A. (2017). Children and young people’s rights in the digital age: An emerging agenda. New Media Soc..

[9] Harvey A., Watkins E., Mansell W., Shafran R. (2004). Cognitive Behavioural Processes across Psychological Disorders: A Transdiagnostic Approach to Research and Treatment.

[10] Crocker L.D., Heller W., Warren S.L., O’Hare A.J., Infantolino Z.P., Miller G.A. (2013). Relationships among cognition, emotion, and motivation: Implications for intervention and neuroplasticity in psychopathology. Front. Hum. Neurosci..

[11] Tay L., Jebb A.T. (2018). Establishing construct continua in construct validation: The process of continuum specification. Adv. Methods Pract. Psychol. Sci..

[12] Fuchs C., Hofkirchner W., Schafranek M., Raffl C., Sandoval M., Bichler R. (2010). Theoretical foundations of the web: Cognition, communication, and co-operation. Towards an understanding of web 1.0, 2.0, 3.0. Future Internet.

[13] Andrejevic M. (2002). The work of being watched: Interactive media and the exploitation of self-disclosure. Crit. Stud. Media Commun..

[14] Utz S. (2015). The function of self-disclosure on social network sites: Not only intimate, but also positive and entertaining self-disclosures increase the feeling of connection. Comput. Hum. Behav..

[15] Kashian N., Jang J., Shin S.Y., Dai Y., Walther J.B. (2017). Self-disclosure and liking in computer-mediated communication. Comput. Hum. Behav..

[16] Oh H.J., Ozkaya E., LaRose R. (2014). How does online social networking enhance life satisfaction? The relationships among online supportive interaction, affect, perceived social support, sense of community, and life satisfaction. Comput. Hum. Behav..

[17] Kuss D., Harkin L., Kanjo E., Billieux J. (2018). Problematic smartphone use: Investigating contemporary experiences using a convergent design. Int. J. Environ. Res. Public Health.

[18] Osatuyi B., Turel O. (2018). Tug of war between social self-regulation and habit: Explaining the experience of momentary social media addiction symptoms. Comput. Hum. Behav..

[19] Buglass S.L., Binder J.F., Betts L.R., Underwood J.D.M. (2017). Motivators of online vulnerability: The impact of social network site use and FOMO. Comput. Hum. Behav..

[20] Pontes H.M., Taylor M., Stavropoulos V. (2018). Beyond “Facebook addiction”: The role of cognitive-related factors and psychiatric distress in social networking site addiction. Cyberpsychol. Behav. Soc. Netw..

[21] Konok V., Pogány Á., Miklósi Á. (2017). Mobile attachment: Separation from the mobile phone induces physiological and behavioural stress and attentional bias to separation-related stimuli. Comput. Hum. Behav..

[22] David-Ferdon C., Hertz M.F. (2009). Electronic Media and Youth Violence: A CDC Issue Brief for Researchers.

[23] Suler J.R. (2016). Psychology of the digital age. Humans Become Electric.

[24] Groshek J., Cutino C. (2016). Meaner on mobile: Incivility and impoliteness in communicating contentious politics on sociotechnical networks. Soc. Media Soc..

[25] Kumar R., Ojha A.K., Malmasi S., Zampieri M. Benchmarking aggression: Identification in social media. Proceedings of the First Workshop on Trolling, Aggression and Cyberbullying.

[26] Hardaker C. (2010). Trolling in asynchronous computer-mediated communication: From user discussions to academic definitions. J. Politeness Res. Lang. Behav. Cult..

[27] Whittle J., Elder-Vass D., Lumsden K., Lumsden K., Harmer E. (2019). ‘There’s a bit of banter’: How male teenagers ‘Do boy’ on social networking sites. Online Othering: Exploring Digital Violence and Discrimination on the Web.

[28] Vandebosch H., Van Cleemput K. (2008). Defining cyberbullying: A qualitative research into the perceptions of youngsters. CyberPsychol. Behav..

[29] Kuss D.J., Griffiths M.D. (2017). Social networking sites and addiction: Ten lessons learned. Int. J. Environ. Res. Public Health.

[30] Mills K.L. (2014). Effects of Internet use on the adolescent brain: Despite popular claims, experimental evidence remains scarce. Trends Cogn. Sci..

[31] Fink E., Patalay P., Sharpe H., Holley S., Deighton J., Wolpert M. (2015). Mental health difficulties in early adolescence: A comparison of two cross-sectional studies in England from 2009 to 2014. J. Adolesc. Health.

[32] Foulkes L., Blakemore S.-J. (2016). Is there heightened sensitivity to social reward in adolescence?. Curr. Opin. Neurobiol..

[33] Mitchell L., Hussain Z. (2018). Predictors of problematic smartphone use: An examination of the integrative pathways model and the role of age, gender, impulsiveness, excessive reassurance seeking, extraversion, and depression. Behav. Sci..

[34] Glaser B.G., Strauss A.L. (2006). The Discovery of Grounded Theory: Strategies for Qualitative Research.

[35] Strauss A., Corbin J.M. (1990). Basics of Qualitative Research: Grounded Theory Procedures and Techniques.

[36] Charmaz K. (2006). Constructing Grounded Theory.

[37] Mills J., Bonner A., Francis K. (2006). The development of constructivist grounded theory. Int. J. Qual. Methods.

[38] Charmaz K. (2017). Constructivist grounded theory. J. Posit. Psychol..

[39] Helms S.W., Choukas-Bradley S., Widman L., Giletta M., Cohen G.L., Prinstein M.J. (2014). Adolescents misperceive and are influenced by high-status peers’ health risk, deviant, and adaptive behavior. Dev. Psychol..

[40] Kuss D.J., Griffiths M.D. (2011). Online social networking and addiction—A review of the psychological literature. Int. J. Environ. Res. Public Health.

[41] Yurdagül C., Kircaburun K., Emirtekin E., Wang P., Griffiths M.D. (2019). Psychopathological consequences related to problematic Instagram use among adolescents: The mediating role of body image dissatisfaction and moderating role of gender. Int. J. Ment. Health Addict..

[42] Emirtekin E., Balta S., Sural I., Kircaburun K., Griffiths M.D., Billieux J. (2019). The role of childhood emotional maltreatment and body image dissatisfaction in problematic smartphone use among adolescents. Psychiatry Res..

[43] Kircaburun K., Griffiths M.D., Billieux J. (2019). Childhood emotional maltreatment and problematic social media use among adolescents: The mediating role of body image dissatisfaction. Int. J. Ment. Health Addict..

[44] Reinecke L., Klimmt C., Meier A., Reich S., Hefner D., Knop-Huelss K., Rieger D., Vorderer P. (2018). Permanently online and permanently connected: Development and validation of the Online Vigilance Scale. PLoS ONE.

[45] Döring N. (2014). Consensual sexting among adolescents: Risk prevention through abstinence education or safer sexting?. Cyberpsychol. J. Psychosoc. Res. Cyberspace.

[46] Festinger L. (1954). A Theory of Social Comparison Processes. Hum. Relat..

[47] Moyal N. (2014). Cognitive strategies to regulate emotions—Current evidence and future directions. Front. Psychol..

[48] Wolgast M., Lundh L.-G. (2017). Is distraction an adaptive or maladaptive strategy for emotion regulation? A person-oriented approach. J. Psychopathol. Behav. Assess..

[49] Lowry P.B., Zhang J., Wang C., Siponen M. (2016). Why do adults engage in cyberbullying on social media? An integration of online disinhibition and deindividuation effects with the social structure and social learning model. Inf. Syst. Res..

[50] Chotpitayasunondh V., Douglas K.M. (2016). How “phubbing” becomes the norm: The antecedents and consequences of snubbing via smartphone. Comput. Hum. Behav..

[51] Freedman G., Williams K.D., Beer J.S. (2016). Softening the blow of social exclusion: The responsive theory of social exclusion. Front. Psychol..

[52] Bayer J.B., LaRose R., Verplanken B. (2018). Technology habits: Progress, problems, and prospects. The Psychology of Habit.

[53] Clayton R.B., Leshner G., Almond A. (2015). The extended iSelf: The impact of iPhone separation on cognition, emotion, and physiology. J. Comput. Mediat. Commun..

[54] Han S., Kim K.J., Kim J.H. (2017). Understanding nomophobia: Structural equation modelling and semantic network analysis of smartphone separation anxiety. Cyberpsychol. Behav. Soc. Netw..

[55] Cheever N.A., Rosen L.D., Carrier L.M., Chavez A. (2014). Out of sight is not out of mind: The impact of restricting wireless mobile device use on anxiety levels among low, moderate and high users. Comput. Hum. Behav..

[56] Johannes N., Veling H., Dora J., Meier A., Reinecke L., Buidjen M. (2018). Mind-wandering and mindfulness as mediators of the relation between online vigilance and well-being. CyberPsychol Behav. Soc. Netw..

[57] Barry C.T., Sidoti C.L., Briggs S.M., Reiter S.R., Lindsey R.A. (2017). Adolescent social media use and mental health from adolescent and parent perspectives. J. Adolesc..

[58] Gazzaley A., Rosen L.D. (2016). The Distracted Mind: Ancient Brains in A High-Tech World.

[59] Webb T.L., Miles E., Sheeran P. (2012). Dealing with feeling: A meta-analysis of the effectiveness of strategies derived from the process model of emotion regulation. Psychol. Bull..

[60] Nolen-Hoeksema S., Wisco B.E., Lyubomirsky S. (2008). Rethinking rumination. Perspect. Psychol. Sci..

[61] Baron R.S., Berkowitz L. (1986). Distraction-conflict theory: Progress and problems. Advances in Experimental Social Psychology.

[62] Baron R.S., Moore D., Sanders G.S. (1978). Distraction as a source of drive in social facilitation research. J. Personal. Soc. Psychol..

[63] Thornton B., Faires A., Robbins M., Rollins E. (2014). The mere presence of a cell phone may be distracting: Implications for attention and task performance. Soc. Psychol..

[64] Triplett N. (1898). The dynamogenic factors in pacemaking and competition. Am. J. Psychol..

[65] Zimbardo P.G., Arnold W.D., Levin D. (1969). The human choice: Individuation reason and order versus deindividuation impulse and chaos. Nebraska Symposium on Motivation.

[66] Rosenberg J., Egbert N. (2011). Online impression management: Personality traits and concerns for secondary goals as predictors of self-presentation tactics on Facebook. J. Comput. Mediat. Commun..

[67] Prentice-Dunn S., Rogers R.W. (1982). Effects of public and private self-awareness on deindividuation and aggression. J. Personal. Soc. Psychol..

[68] Pryor C., Perfors A., Howe P.D.L. (2019). Even arbitrary norms influence moral decision-making. Nat. Hum. Behav..

[69] Twenge J.M., Spitzberg B.H., Campbell W.K. (2019). Less in-person social interaction with peers among U.S. adolescents in the 21st century and links to loneliness. J. Soc. Pers. Relatsh..

[70] Kircaburun K., Demetrovics Z., Király O., Griffiths M.D. (2019). Childhood emotional trauma and cyberbullying perpetration among emerging adults: A multiple mediation model of the role of problematic social media use and psychopathology. Int. J. Ment. Health Addict..

[71] Karadağ E., Tosuntaş Ş.B., Erzen E., Duru P., Bostan N., Şahin B.M., Çulha İ., Babadağ B. (2015). Determinants of phubbing, which is the sum of many virtual addictions: A structural equation model. J. Behav. Addict..

[72] Balta S., Emirtekin E., Kircaburun K., Griffiths M.D. (2019). Neuroticism, trait fear of missing out, and phubbing: The mediating role of state fear of missing out and problematic Instagram use. Int. J. Ment. Health Addict..

[73] Franchina V., Vanden Abeele M., van Rooij A., Lo Coco G., De Marez L. (2018). Fear of missing out as a predictor of problematic social media use and phubbing behavior among Flemish adolescents. Int. J. Environ. Res. Public Health.

[74] Kelly Y., Zilanawala A., Booker C., Sacker A. (2019). Social media use and adolescent mental health: Findings from the UK Millennium cohort study. EClinicalMedicine.

[75] Dibb B. (2019). Social media use and perceptions of physical health. Heliyon.

[76] Fardouly J., Diedrichs P.C., Vartanian L.R., Halliwell E. (2015). Social comparisons on social media: The impact of Facebook on young women’s body image concerns and mood. Body Image.

[77] Holland G., Tiggemann M. (2016). A systematic review of the impact of the use of social networking sites on body image and disordered eating outcomes. Body Image.

[78] Reer F., Tang W.Y., Quandt T. (2019). Psychosocial well-being and social media engagement: The mediating roles of social comparison orientation and fear of missing out. New Media Soc..

[79] Balakrishnan J., Griffiths M.D. (2018). An exploratory study of “selfitis” and the development of the Selfitis Behavior Scale. Int. J. Ment. Health Addict..

[80] Bij de Vaate A.J.D., Veldhuis J., Alleva J.M., Konijn E.A., van Hugten C.H.M. (2018). Show your best self(ie): An exploratory study on selfie-related motivations and behavior in emerging adulthood. Telemat. Inform..

[81] Madigan S., Ly A., Rash C.L., Van Ouytsel J., Temple J.R. (2018). Prevalence of multiple forms of sexting behavior among youth: A systematic review and meta-analysis. JAMA Pediatr..

[82] Tokunaga R.S. (2011). Social networking site or social surveillance site? Understanding the use of interpersonal electronic surveillance in romantic relationships. Comput. Hum. Behav..

[83] Brown Z., Tiggemann M. (2016). Attractive celebrity and peer images on Instagram: Effect on women’s mood and body image. Body Image.

[84] Alter A. (2017). Irresistible: The Rise of Addictive Technology and the Business of Keeping Us Hooked.

[85] Lew Z., Walther J.B., Pang A., Shin W. (2018). Interactivity in online chat: Conversational contingency and response latency in computer-mediated communication. J. Comput. Mediat. Commun..

[86] Lally P., Gardner B. (2013). Promoting habit formation. Health Psychol. Rev..

[87] Tiggemann M., Barbato I. (2018). “You look great!”: The effect of viewing appearance-related Instagram comments on women’s body image. Body Image.

[88] Frison E., Eggermont S. (2017). Browsing, posting, and liking on Instagram: The reciprocal relationships between different types of Instagram use and adolescents’ depressed mood. Cyberpsychol. Behav. Soc. Netw..

[89] Vargas T., Maloney J., Gupta T., Damme K.S.F., Kelley N.J., Mittal V.A. (2019). Measuring facets of reward sensitivity, Inhibition, and impulse control in individuals with problematic internet use. Psychiatry Res..

[90] Sherman L.E., Payton A.A., Hernandez L.M., Greenfield P.M., Dapretto M. (2016). The power of the ‘like’ in adolescence: Effects of peer influence on neural and behavioral responses to social media. Psychol. Sci..

[91] Andreassen C.S., Pallessen S. (2014). Social network site addiction—An overview. Curr. Pharm. Des..

[92] Throuvala M.A., Griffiths M.D., Rennoldson M., Kuss D.J. (2019). School-based prevention for adolescent internet addiction: Prevention is the key. A systematic literature review. Curr. Neuropharmacol..

[93] Vallejos E.P., Siebers P.-O., Craven M., Nilsson T., Siebert P., Fuentes C. Untangling multi-stakeholder perspectives in digital mental healthcare. Proceedings of the 4th Symposium on Computing and Mental Health.

[94] Griffin A. (2019). Instagram to try banning likes everywhere. The Independent.

[95] Bonello M., Griffiths M.D. (2019). Behavioural tracking, responsible gambling tools and online voluntary self-exclusion: Implications for problem gamblers. Casino Gambl. Int..

